# Facile preparation of multifunctional biomaterials BTO/Ag and their applications in photoelectrochemical sensing, photodegradation and antibacterial activities†

**DOI:** 10.1039/d4ra07385a

**Published:** 2025-01-27

**Authors:** Zhang Kexi, Yan Bingdong, Chen Delun, Wang Xiaohong, Cao Yang, Zhang Xuewei, Hao Wanjun, Tu Jinchun

**Affiliations:** a College of Material Science and Engineering, Key Laboratory of Advanced Materials of Tropical Island Resources, Ministry of Education, Hainan University Haikou 570228 China zhangxuewei@hainanu.edu.cn hwj8899@hainanu.edu.cn tujinchun@hainanu.edu.cn; b Key Laboratory of Child Cognition and Behavior Development of Hainan Province, Qiongtai Normal University Haikou 571100 China

## Abstract

With the progress of modern technology and the diversification of societal demands, traditional materials with single properties can no longer meet the requirements of complex and constantly evolving application scenarios. To tackle intricate biomedical applications like disease diagnosis and treatment, scientists are focusing on exploring the design of novel multifunctional biomaterials that possess diverse activities. Bismuth titanate (Bi_4_Ti_3_O_12_, BTO), which has multifunctionality and great application potential, unfortunately suffers from inadequate photocatalytic performance. On the other hand, silver nanoparticles (Ag), known for their antibacterial properties, have relatively limited functions. In this study, we overcame these limitations by combining BTO with Ag to form a BTO/Ag biomultifunctional material. Our experiments showed that the addition of Ag effectively improved BTO's UV absorption ability, decreased electron transfer resistance, and increased carrier concentration. As a result, the photocatalytic performance of BTO/Ag was significantly enhanced, and its photoelectrochemical sensing and photodegradation capabilities were also greatly improved. Moreover, BTO served as an effective substrate, preventing Ag from agglomerating and maximizing its antibacterial potential. In specific performance evaluations, ascorbic acid and methylene blue (MB) were used to study the photoelectrochemical sensing and photodegradation capabilities respectively, while *Escherichia coli* and *Staphylococcus aureus* were chosen as test organisms to assess the antibacterial properties. All in all, this research has yielded promising results.

## Abstract 2

With the progress of modern technology and the diversification of societal demands, traditional materials with single properties can no longer meet the requirements of complex and constantly evolving application scenarios.

## Graphical abstract

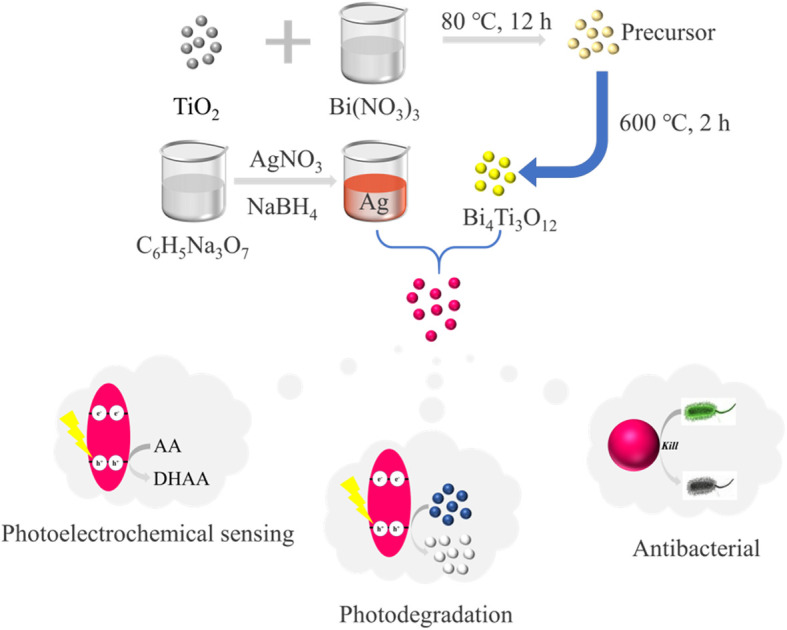

## 1 Introduction

Driven by the rapid development of modern technology and the diversification of social needs, traditional materials with single performance have been unable to meet the complex and varied application scenarios. As life sciences, materials science, and medical engineering converge and advance rapidly, scientists are exploring the design and preparation of new materials that possess biocompatibility, biodegradability, and multiple bioactivities. These materials are intended to meet the complex demands of biomedical applications such as disease diagnosis and treatment. In this context, the emergence of multifunctional biomaterials has become one of the hotspots in materials science research.

Research into bio-multifunctional materials has shown rapid progress in the realms of sensing and antibacterial applications, showcasing their potential for widespread use. In sensing, due to their unique biocompatibility, high sensitivity, and specific recognition capabilities, bio-multifunctional materials have been crucial in the construction of high-performance biosensors.^1–4^ These materials have proven adept at precisely detecting various physiological parameters and biomarkers within biological organisms, thereby providing strong support for early disease diagnosis, treatment monitoring, and health management. Similarly, in the field of antibacterial research, bio-multifunctional materials have also demonstrated immense potential. Researchers have engineered antibacterial biomaterials,^5–8^ including composites loaded with antibacterial agents and surface materials modified with antibacterial peptides, which have been effective in curbing bacterial growth and reproduction, reducing the risk of tissue infections. As understanding of antibacterial mechanisms continue to evolve, the application prospects of bio-multifunctional materials in the antibacterial field are expected to expand significantly.

In recent years, perovskite has demonstrated immense potential in various fields.^9,10^ The Aurivillius structure, a type of layered perovskite oxide structure, is distinguished by its unique characteristics and advantages.^11^ It consists of alternating stacks of perovskite layers and bismuth oxide layers, forming a distinctive layered configuration. The robust chemical bonds between the perovskite layers and bismuth oxide layers in the Aurivillius structure confer high chemical stability, allowing Aurivillius-phase materials to perform reliably under various environmental conditions and exhibit potential applications in electrical,^12^ magnetic,^13^ optical,^14^ and catalytic fields.^15^ Bismuth titanate (Bi_4_Ti_3_O_12_, BTO), a typical Aurivillius-phase material,^16^ boasts excellent thermal and chemical stability, ample room for performance optimization, and multifunctionality, thereby presenting broad application prospects and development potential across multiple domains. Nevertheless, when utilizing BTO's photocatalytic properties to construct biomultifunctional materials for sensing and photodegradation, several challenges arises,^17–19^ such as low visible light absorption capacity, high photogenerated electron–hole recombination rate, and the occurrence of photocorrosion. To overcome these limitations, researchers have been actively exploring novel synthesis methods and modification strategies to enhance BTO's photocatalytic performance and its application value.

On the other hand, Ag, as a common antibacterial material, has been extensively researched.^20–23^ Silver nanoparticles, due to their unique particle size and surface properties, can efficiently contact and kill over 650 types of bacteria and viruses, including *Escherichia coli*, *Staphylococcus aureus*, *Candida albicans*, *Pseudomonas aeruginosa*, *Bacillus subtilis*, and *Aspergillus niger*, at extremely low concentrations (ranging from a few ppb to a few ppm), without inducing drug resistance. Reports^24–26^ indicate that silver nanoparticles primarily achieve this by releasing silver ions, which interfere with cell wall synthesis, damage cell membranes, inhibit protein synthesis, disrupt nucleic acid synthesis, and react with sulfur-containing hydride groups (–SH) in bacteria, ultimately leading to microbial inactivation and death. Furthermore, Ag has been utilized as a photosensitizing material, effectively enhancing the overall performance of photocatalytic materials and demonstrating unique application potential in fields such as photocatalysis. This dual functionality of Ag, in both antibacterial and photocatalytic applications, underscores its value in the development of multifunctional materials.

While both BTO and Ag face challenges when used alone as multifunctional biomaterials due to their inherent limitations, their integration has been found to significantly enhance the overall properties of the resultant material. Firstly, as a metal, Ag is able to modulate the electronic and band structures of BTO, thereby broadening its light absorption range to include visible and even infrared light, leading to improved solar energy utilization. Secondly, with a high work function, Ag serves as an efficient electron trap, effectively inhibiting the recombination of electrons and holes, thus enhancing the separation efficiency of photogenerated carriers in BTO. Lastly, the surface modification by Ag increase the activity of photocatalytic reactions. Concurrently, BTO, serving as the support for Ag, facilitates the effective dispersion of Ag, reducing its consumption and lowering the material's cost. The combination of Ag and BTO forms a stable structure that minimizes the loss and valence change of Ag, thereby enhancing the durability of its antibacterial effects. In summary, the composite of BTO and Ag is expected to yield a superior multifunctional biomaterial, with comprehensively improved photocatalytic and antibacterial performances, poised for effective applications in fields such as photoelectric sensors, photodegradation, and antibacterial treatment.

**Scheme 1:**
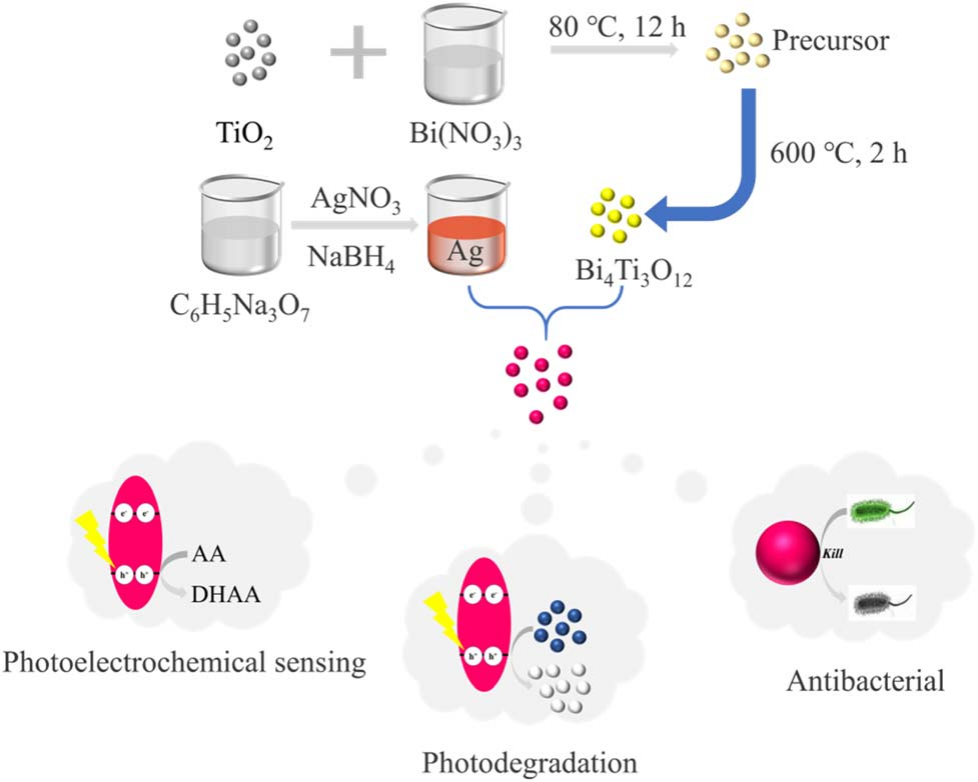
Synthesis and multifunctional application of BTO/Ag.

In the pursuit of superior-performing multifunctional biomaterials, this study combined BTO with Ag to construct BTO/Ag (Scheme 1). In the investigation of photoelectrochemical sensing and photodegradation properties, it was discovered that Ag effectively optimized BTO's UV absorption characteristics. Diminished electron transfer resistance, and enhanced carrier concentration. These improvements consequently boosted the photocatalytic performance of BTO/Ag and, in turn, bolstering its photoelectrochemical sensing and photodegradation capabilities. Regarding antibacterial performance, it was found that BTO served as an excellent support for Ag, preventing its agglomeration to fully exhibit its antibacterial properties. In specific performance evaluations, ascorbic acid was utilized for assessing photoelectrochemical sensing properties, MB was employed for examining photodegradation properties, and *Escherichia coli* and *Staphylococcus aureus* were chosen as representatives of Gram-negative and Gram-positive bacteria, respectively, for antibacterial performance studies.

## 2 Experimental sections

### 2.1 Preparation of BTO

0.4 g TiO_2_ was added to 20 mL 3 M bismuth nitrate solution, followed by 15 min sonication dispersion. It was then aged at room temperature for 12 hours and filtered. The obtained sample was then dried at 80 °C for 12 hours to yield the precursor. The precursor was placed in a tube furnace, which was heated at a rate of 2 °C per minute until the temperature reached 600 °C, and held there for 2 hours. Afterward, it was naturally cooled down to room temperature, resulting in BTO.

### 2.2 Preparation of Ag nanoparticles

20 mL of a 1 wt% trisodium citrate dihydrate solution was mixed with 75 mL distilled water and heated to 70 °C, followed by continuous stirring for 15 minutes. Subsequently, 1.7 mL of a 1 wt% silver nitrate solution and 2 mL of 0.1 wt% freshly prepared sodium borohydride solution were added to the mixture. The resulting mixture was vigorously stirred at 70 °C for 1 hour, allowed to cool to room temperature, and then diluted to a final volume of 100 mL.

### 2.3 Preparation of BTO/Ag

0.25 g polyethyleneimine (PEI) was added to 50 mL distilled water and sonicated for 10 minutes. Subsequently, 0.2 g BTO was added and sonicated for 2 hours, followed by centrifugation and washing. The obtained product was redispersed in 10 mL of distilled water, and then 10 mL of a Ag nanoparticle solution (0.108%, estimate based on the feeding ratio) was added. The mixture was sonicated for 1 hour, centrifuged and washed, resulting in the acquisition of BTO/Ag.

### 2.4 Characterization

The sample for XRD was carefully ground. The samples for SEM and TEM were ultrasonically dispersed and drop-coated on the sample stage. The electrochemical test was carried out in a buffer solution using a three-electrode system, with a platinum electrode as the counter electrode and an Ag/AgCl electrode as the reference electrode.

### 2.5 Sensing, photocatalytic and antibacterial assays

The sensor was constructed using a three-electrode system, and the test solution was PBS buffer, with the test carried out at room temperature. The medium for the photocatalytic experiment was distilled water, and the light source used was simulated sunlight. Before photodegradation, a light-proof treatment was carried out, and the test process was continuously stirred. The antibacterial experiment was conducted using LB solid medium.

All the data given in the article are based on the statistical results of five parallel experiments. The synthesis yields (mass per mass, in %) for each material were obtained by analyzing the results of five replicate experiments. For BTO, its synthesis yields was 91.3% and the standard deviation was 5.2%. For BTO/Ag, its synthesis yields was 92.6% and the standard deviation was 6.8%. It is worth noting that since this reaction mainly involves the transformation among inorganic substances and there are relatively few side reactions, the synthesis yields being lower than 100% are mainly due to the losses caused by the sample washing process. However, for Ag nanoparticles, we used an excessive amount of reducing agent to reduce Ag^+^, and the obtained samples were not subjected to any treatment. Therefore, the synthesis yields of Ag was defined as 100%.

## 3 Results and discussion

**Fig. 1:**
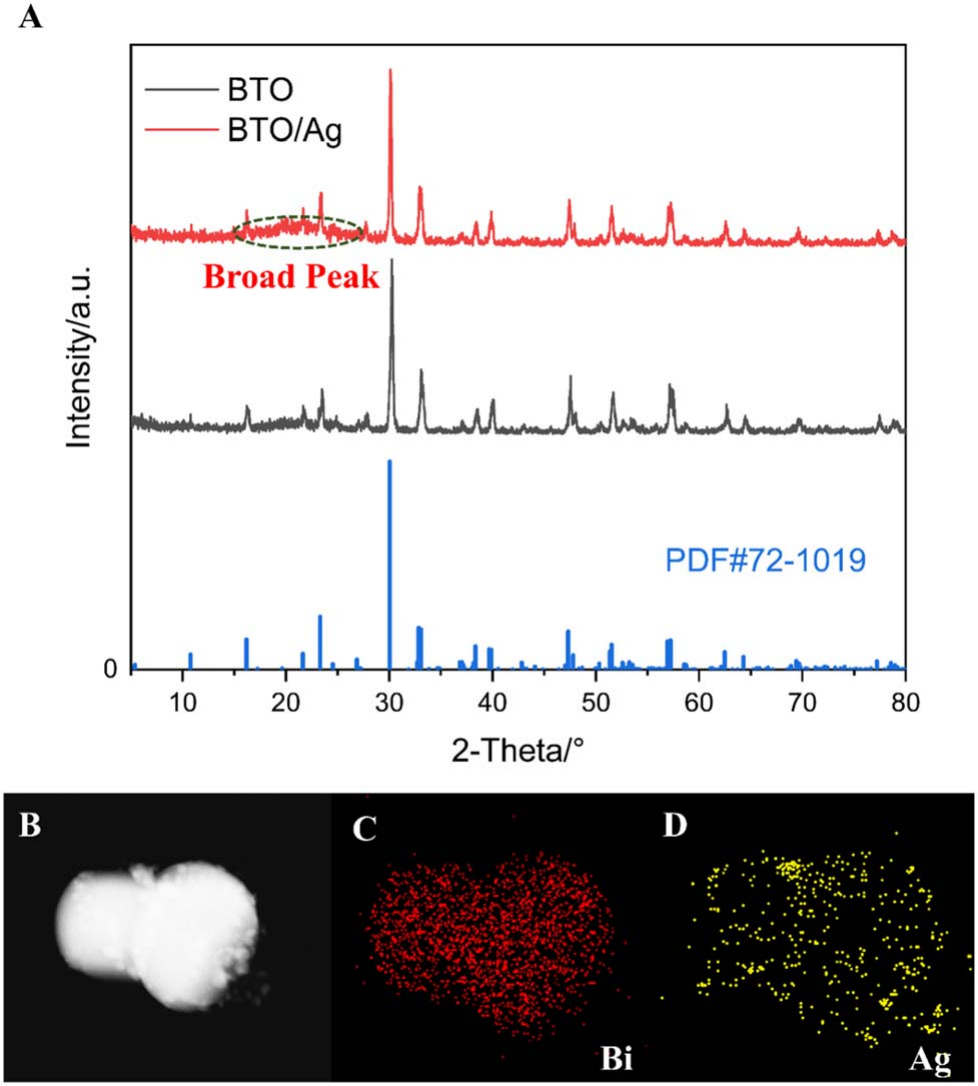
(A) XRD for BTO and BTO/Ag; SEM of (B) BTO/Ag; mapping of (C) Bi and (D) Ag in BTO/Ag.

XRD was utilized to investigate the crystalline phases of BTO/Ag, with the results presented in Fig. 1A. The XRD analysis confirmed that the primary crystalline phase of both BTO and BTO/Ag was Bi_4_Ti_3_O_12_ (PDF #72-1019), indicating that the crystalline phase of the material remained largely unchanged after the loading of Ag. Nevertheless, between 10° to 25°, a relatively weak and broadened peak emerged in the XRD pattern of BTO/Ag, which could be attributed to the small particle size of Ag nanoparticles and the use of PEI as a linker during the loading process. To further confirm the presence of Ag, EDS analysis was conducted, with the results shown in Fig. 1B–D. The elemental mapping results for Bi and Ag revealed a high degree of spatial overlap, suggesting that Ag was successfully loaded onto the surface of BTO and achieved a uniform dispersion.

**Fig. 2:**
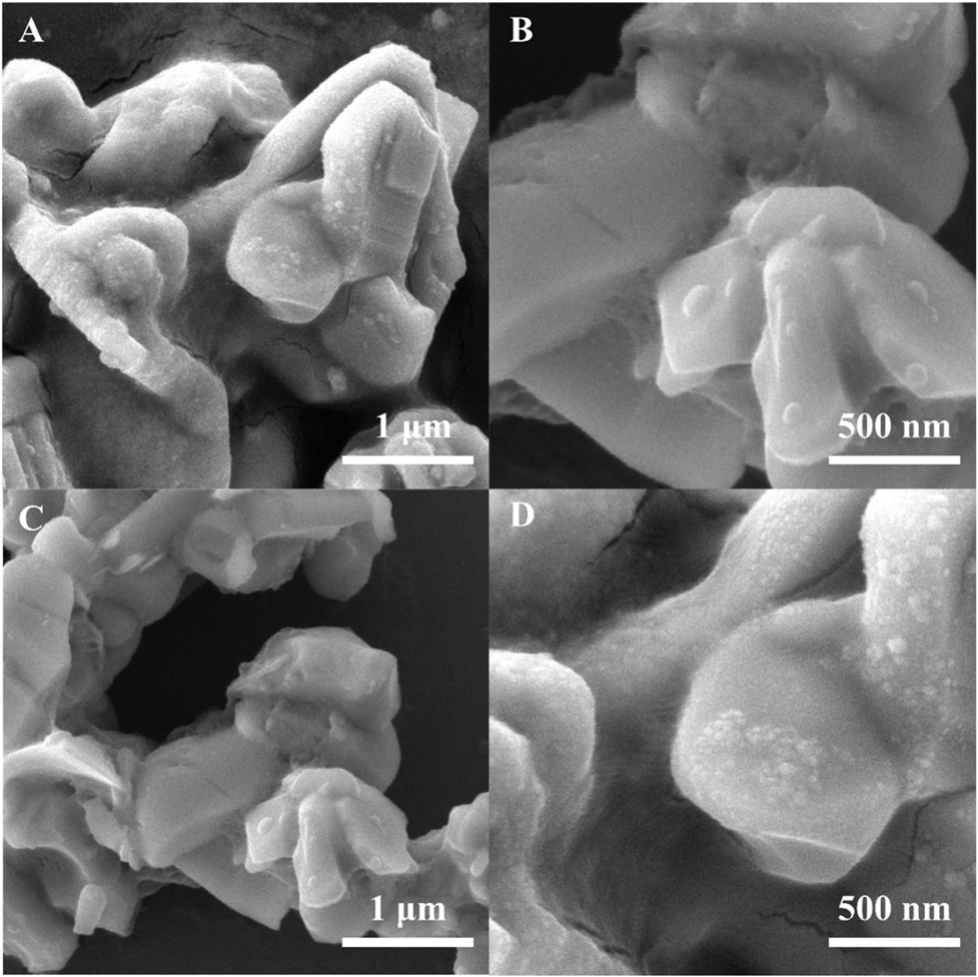
SEM of (A and B) BTO and (C and D) BTO/Ag.

To analyze the morphology of BTO/Ag, SEM was employed and the results are presented in Fig. 2. Fig. 2A and B depict the morphology of BTO, while Fig. 2C and D show the morphology of BTO/Ag. According to these results, the experimentally obtained BTO exhibits an irregular particle shape, composed of numerous smaller particles. Compared to BTO, the morphology of BTO/Ag does not undergo significant changes, except for a rougher surface, which is likely attributed to the presence of Ag nanoparticles on the BTO surface. As displayed in Fig. 2D, the Ag particles are relatively small, with sizes less than 10 nm. The use of PEI enables Ag to be well-anchored on the surface of BTO while maintaining good monodispersity. In addition, the particle sizes for each material could be estimated as follow: the particle size of BTO was irregular particles with a size of 2 μm ± 300 nm. The particle size of Ag was 50 nm ± 20 nm, which might be caused by the agglomeration of Ag. In BTO/Ag, the particle size of Ag was 8 nm ± 2 nm, indicating that BTO was conducive to the dispersion of Ag nanoparticles. In addition, the size of BTO/Ag was similar to that of the BTO particles.

**Fig. 3:**
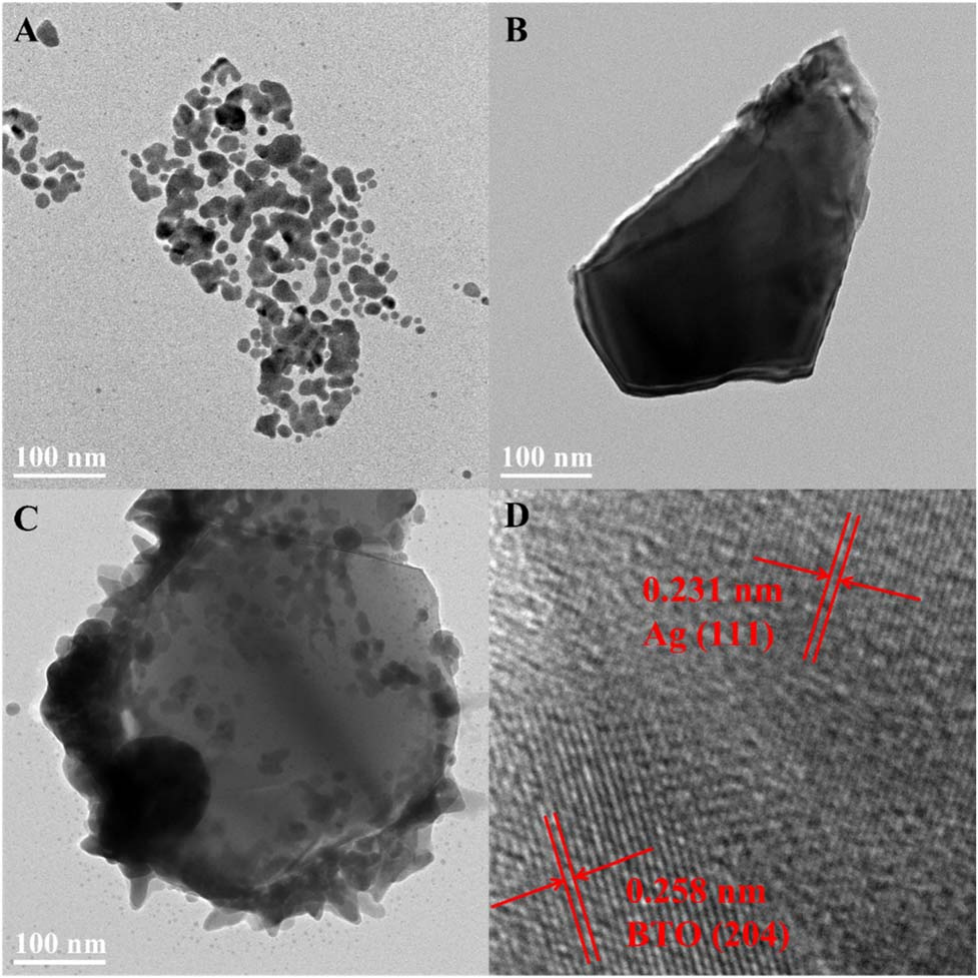
TEM of (A) Ag, (B) BTO, and (C) BTO/Ag; (D) HRTEM of BTO/Ag.

To delve deeper into the morphology of the samples, TEM was employed. Fig. 3A, B and C present the TEM test results of Ag nanoparticles, BTO, and BTO/Ag, respectively. Fig. 3D shows the HRTEM of BTO/Ag with crystal plane identification for further metallographic confirmation. As shown in Fig. 3A, the Ag nanoparticles are primarily spherical but exhibit significant agglomeration. Fig. 3B further confirms that BTO exhibits an irregular particle morphology. The test results of BTO/Ag (Fig. 3C) reveal that Ag is well-dispersed on the surface of BTO. Compared to Fig. 3C, the agglomeration of Ag is significantly mitigated, which effectively increases the specific surface area of Ag, contributing to the enhancement of the overall performance of the material. The HRTEM of BTO/Ag reveals two distinct interplanar spacings: 0.258 nm, which corresponds to the (204) crystal plane of BTO (PDF #72-1019), and 0.231 nm, which corresponds to the (111) crystal plane of Ag (PDF #87-0720). This further demonstrates the successful loading of Ag on the surface of BTO.

**Fig. 4:**
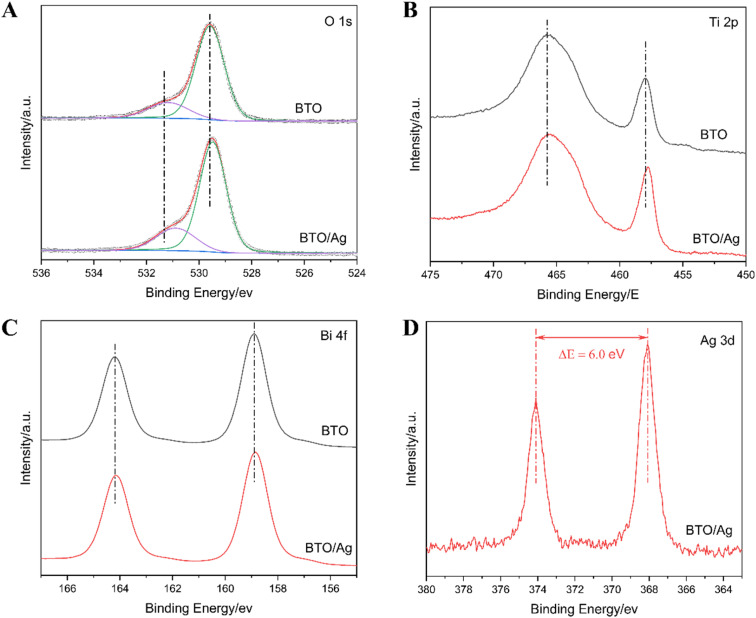
XPS of (A) O 1s, (B) Ti 2p, (C) Bi 4f, and (D) Ag 3d.

To investigate the chemical environment of elements in BTO and their interactions with Ag, XPS was employed to characterize both BTO and BTO/Ag, with the results presented in Fig. 4 and S1.† According to the test results of X-ray photoelectron spectroscopy (XPS), in BTO/Ag, the atomic percentages of Ag, Bi, and Ti are 3.2%, 62.3% and 34.5% respectively; in BTO, the atomic percentages of Bi and Ti are 64.9% and 35.1% respectively. This result is inconsistent with the ratio of Bi to Ti in BTO, which may be caused by the existence of defects in the material. Additionally, based on this result, it can be estimated that the mass fraction of Ag in BTO/Ag is approximately 2.65%. Initially, the XPS spectrum of O 1s (Fig. 4A) was analyzed, showing signal peaks near 531 eV and 529 eV in the O 1s spectrum corresponding to defect oxygen and lattice oxygen in BTO, respectively. Upon addition of Ag, the O 1s peak of BTO/Ag exhibited a redshift, indicating an increase in the electron density of O on BTO. This signifies that Ag acts as electron donor in BTO/Ag, with electrons migrating from Ag to the oxygen in BTO, which can potentially facilitate the conversion of Ag to Ag^+^, accelerate Ag dissolution, and consequently enhance the antibacterial properties of the material. Further studies revealed that defect oxygen accounted for 21.7% of the total oxygen content in BTO, whereas its proportion decreased to 18.2% in BTO/Ag. This decrease suggests that the incorporation of Ag can effectively reduce oxygen defects in BTO, which in turn diminishes scattering during electron migration and thus benefits the acceleration of electron transfer kinetics.

Upon comparing the XPS spectra of BTO and BTO/Ag, no significant changes were observed in the peak shapes of Ti 2p (Fig. 4B) and Bi 4f (Fig. 4C), suggesting that the modification with Ag nanoparticles had little impact on the crystal structure of BTO. However, a pronounced blueshift was noted in the Ti 2p spectrum after modification with Ag nanoparticles, while the Bi 4f spectrum showed a negligible blueshift. This can be attributed to the increased electron density around oxygen atoms due to the presence of Ag nanoparticles (Fig. 4A). This increase in electron density, facilitated by chemical bond conduction, led to an increase in electron density of Ti and Bi atoms, resulting in blueshifts in their XPS spectra. Considering that bismuth titanate consists of alternating layers of perovskite and bismuth oxide, and the blueshift of Ti 2p was more pronounced than that of Bi 4f, it can be inferred that Ag nanoparticles primarily interact with the perovskite layers.

To determine the chemical state of Ag in BTO/Ag, the XPS spectrum of Ag 3d (Fig. 4D) was analyzed. The results show that the Ag 3d spectrum exhibited only two sharp and symmetric peaks with an energy difference of 6.0 eV, indicating a single chemical state of Ag and confirming its presence as a metallic element.^27,28^ Thus, elemental silver exists in the form of silver in BTO/Ag.

**Fig. 5:**
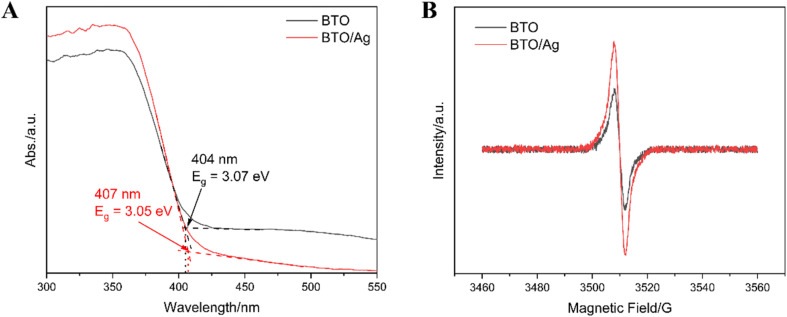
(A) UV-Vis DRS and (B) EPR results of BTO and BTO/Ag.

Ultraviolet-visible diffuse reflectance spectroscopy (UV-Vis DRS) was utilized to investigate the bandgap and absorbance of BTO and BTO/Ag. As depicted in Fig. 5A, the absorption edges of BTO and BTO/Ag were found to be 404 nm and 407 nm, respectively, corresponding to energies of 3.07 eV and 3.05 eV. This result suggests that the bandgap of BTO/Ag is slightly narrower than that of BTO. Additionally, Fig. 5A reveals that BTO/Ag has enhanced light absorption in the ultraviolet region compared to BTO. Its smaller bandgap and superior ultraviolet light absorption confer a higher photoelectric conversion efficiency on BTO/Ag, thereby enhancing its photocatalytic ability.

Electron paramagnetic resonance (EPR) measurements, as presented in Fig. 5B, were conducted to compare the oxygen vacancy concentrations of BTO and BTO/Ag. The test results indicate that BTO/Ag has a higher oxygen vacancy concentration than BTO. Previous studies^29^ suggest that a moderate increase in the oxygen vacancy concentration of a material can favorably enhance its carrier concentration, leading to improved photoelectric properties. Combining the UV-Vis DRS and EPR results, it can be inferred that BTO/Ag, with its strong ultraviolet light absorption, narrower bandgap, and higher oxygen vacancy concentration, exhibits superior photocatalytic performance compared to BTO.

**Fig. 6:**
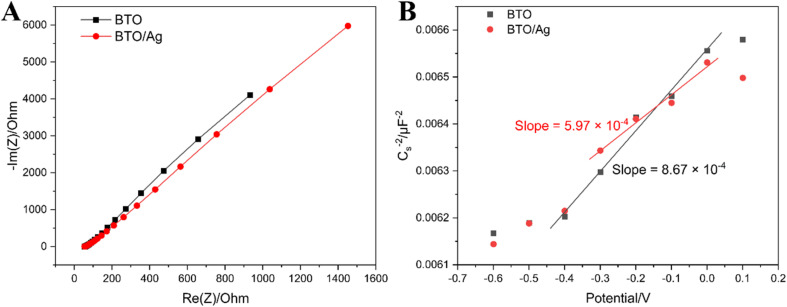
(A) EIS and (B) M–S plot of BTO and BTO/Ag.

Electrochemical impedance spectroscopy (EIS) and Mott–Schottky plot (M–S plot) were utilized to comparatively assess the electron transfer resistance and carrier concentration of BTO and BTO/Ag, thereby providing a comprehensive analysis of the photocatalytic performance of these materials. Fig. 6A clearly shows that the impedance arc radius of BTO/Ag is smaller than that of BTO, which indicates that the modification with Ag effectively reduced the electron transfer resistance of the material, likely attributed to the high conductivity of Ag nanoparticles. The high conductivity of Ag nanoparticles facilitated the transfer of electrons across the BTO surface, subsequently mitigating the accumulation of charges on the surface and enhancing electron transfer efficiency.

To delve into the differences in carrier concentration between BTO and BTO/Ag, further analysis using the M–S plot, as depicted in Fig. 6B, revealed that BTO/Ag exhibited a lower slope in the linear region, suggesting a higher carrier density in BTO/Ag. Given that the carrier density of a material is inversely proportional to its slope, it can be estimated that the carrier density of BTO/Ag is 1.45 times that of BTO, representing an approximately 45% increase in carrier concentration upon modification with Ag nanoparticles. This enhancement is highly beneficial for improving the photocatalytic performance of the material.

In summary, the addition of Ag nanoparticles was found to effectively enhance both the electron transfer capabilities and carrier density of BTO, ultimately leading to a significant improvement in its photocatalytic performance.

Summarizing the results of material characterization, it can be concluded that there are multiple interactions between BTO and Ag. Firstly, BTO can provide an appropriate loading environment for Ag, enabling Ag to have good monodispersity. This is mainly because BTO has specific crystal structures and surface properties. Barium titanate is a ferroelectric material with a perovskite structure. There are abundant surface sites in its crystal structure, and these surface sites can interact with Ag nanoparticles, providing attachment positions for Ag. Meanwhile, the crystal structure of BTO can also restrain the aggregation of Ag nanoparticles, keeping them in good monodispersity.

Secondly, Ag can optimize the electronic structure of BTO, making BTO/Ag have stronger ultraviolet light absorption ability, a smaller bandgap, and a higher oxygen vacancy concentration. The surface plasmon resonance effect of Ag nanoparticles can enhance the ultraviolet light absorption ability of the BTO/Ag composite material. When the frequency of the incident light matches the surface plasmon resonance frequency of Ag nanoparticles, a strong localized electromagnetic field enhancement effect will be generated, thereby improving the absorption of ultraviolet light by the composite material. Meanwhile, the electron transfer between Ag and BTO will lead to a lower bandgap and a higher oxygen vacancy concentration.

Finally, Ag nanoparticles can effectively improve the electron transfer ability and carrier density of BTO, thus achieving an effective improvement in photocatalytic performance. The high electrical conductivity of Ag can promote the electron transfer in BTO and improve the electron transfer efficiency. Meanwhile, the surface plasmon resonance effect of Ag nanoparticles can also excite more electron–hole pairs and increase the carrier density.

**Fig. 7:**
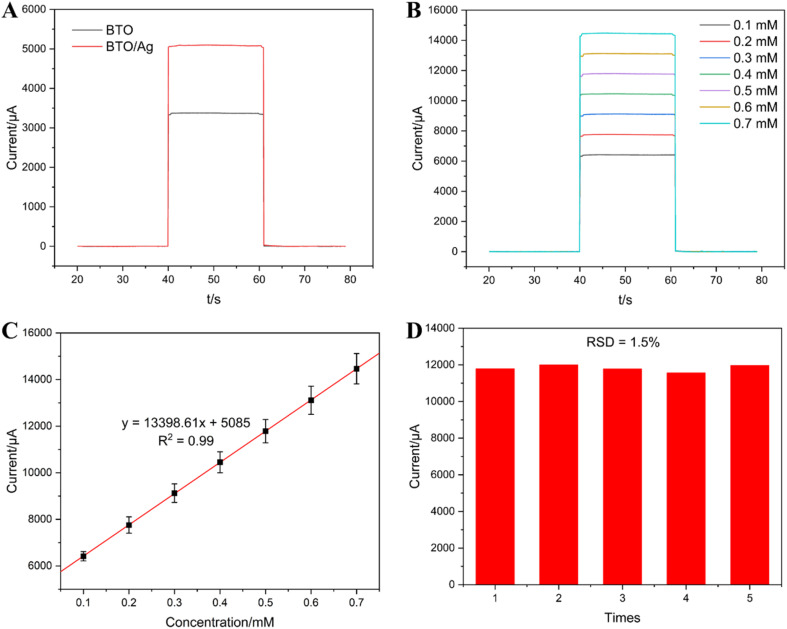
(A) The photoelectric response of BTO and BTO/Ag; (B) the photoelectric response of different concentrations of AA; (C) linear fitting results of different concentrations of AA and photoelectric responses; (D) the results of stability test.

To intuitively investigate the photoelectric properties of BTO and BTO/Ag, the chronoamperometry method was employed in our experiments. Under a potential of 0.3 V, the photocurrent of BTO/Ag (approximately 5000 μA) was significantly higher than that of BTO (approximately 3000 μA), with an increase of 66.7%, demonstrating the superior photoelectric response characteristics of BTO/Ag (Fig. 7A).

Due to its favorable photocatalytic performance, BTO/Ag was further explored in the application research of photoelectric sensors. Ascorbic acid (AA) is a crucial vitamin essential for maintaining a healthy state in the human body. Once the human body is deficient in ascorbic acid, the body's disease resistance ability will decrease significantly, and in severe cases, scurvy can be induced. Ascorbic acid is deeply involved in numerous core biological reaction processes in the human body, covering important aspects such as collagen synthesis, iron absorption, and the growth and repair of cells, tissues, bones, cartilage, teeth, *etc.* It plays a key role in constructing a healthy immune barrier of the body to resist the invasion of bacteria and viruses, and also helps in the synthesis of various chemical messengers and hormones, and occupies an important position in the operation of the human nervous system. In the field of medical identification and diagnosis, achieving rapid, sensitive, and selective detection of ascorbic acid is of extremely important value. As a biomarker, ascorbic acid can be used to identify various abnormal functional conditions in the human body, such as the detection of immune function decline, scurvy, cardiovascular diseases, Alzheimer's disease, Parkinson's disease, and other diseases.

Fig. 7B presents the photoelectric response curves of BTO/Ag under different ascorbic acid (AA) concentrations. The experimental results indicate that as the AA concentration increased, the photoelectric response of BTO/Ag intensified correspondingly, illustrating its excellent response to AA and a clear positive correlation between the response intensity and AA concentration. To further investigate the functional relationship between them, the fitting results of AA concentration and photoelectric response intensity are provided in Fig. 7C. The linear regression coefficient of determination (*R*^2^) between AA concentration and photoelectric response within the concentration range of 0.1 to 0.7 mM was 0.99, confirming a strong linear correlation. This implies that BTO/Ag, when used as the sensing material, has a linear detection range for AA between 0.1 and 0.7 mM. Additionally, the slope of the fitted linear equation indicates a sensitivity of 13 398.61 μA mM^−1^, as the same as 13.4 mA mM^−1^, for BTO/Ag towards AA, which outperforms previous research results (Table 1). Furthermore, based on the fitting equation, under a signal-to-noise ratio of 3 (S/N = 3), the detection limit of BTO/Ag for AA can be estimated to be 1 μM. The results of five repeated experiments, depicted in Fig. 7D, exhibit a relative standard deviation (R.S.D) of 1.5%, indicating good test stability.

**Table 1:** Analytical performance of AA sensor reported in previous work

Material	Sensitivity (μA mM^−1^)	Linear range (mM)	LOD (μM)	Reference
PdNi/C	760.6	N/A	N/A	30
MgO nanobelts	14	0.025–0.150	0.2	31
CoPc–MWNTs	1813.07	0.01–2.6	1	32
RGO/ZnO/GCE	11.9	0.05–2.35	1.12	33
PEDOT-lauroylsarcosinate	80.4	0.002–14	N/A	34
BTO/Ag	13 398.61	0.1–0.7	1	This work

Glucose, uric acid, dopamine hydrochloride, and NaCl as interfering substances to test the anti-interference ability of the sensor, since glucose, uric acid, dopamine, sodium chloride and ascorbic acid often coexist in body fluids, and their signals usually overlap, resulting in interference. The experimental results showed that the response intensities of these substances were 2.1%, 4.3%, 3.8%, and 1.2% of the AA response intensity respectively (Fig. S2†). These data indicate that the electrode has a good anti-interference ability.

**Fig. 8:**
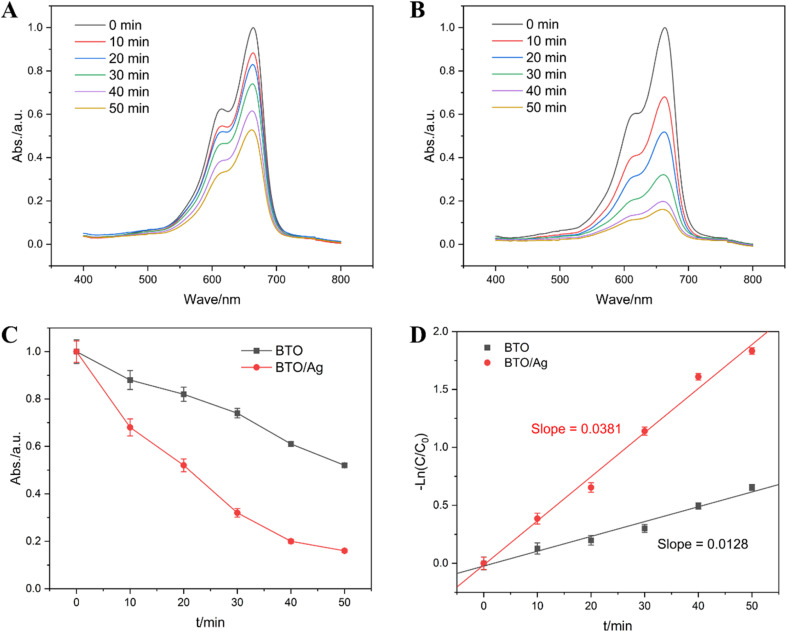
Absorption curves at different times in the presence of (A) BTO and (B) BTO/Ag; the linear fitting results: (C) *t vs.* Abs. and (D) *t vs.* −ln(*C*/*C*_0_).

Recognizing the vital role of high-performance photocatalytic materials in photodegradation, an experimental study was conducted to compare the photodegradation performance of BTO and BTO/Ag against methylene blue (MB). Fig. 8A and B illustrate the changes in absorbance over time when BTO and BTO/Ag were used as photocatalysts, respectively, under UV irradiation, with samples taken and tested every 10 minutes. The maximum absorption peak of MB was identified at 663 nm, and its intensity gradually decreased over time, indicating that both BTO and BTO/Ag exhibited photodegradation performance. A detailed comparison of the results in Fig. 8A and B shows a more significant change over time for BTO/Ag, suggesting its superior photodegradation performance towards MB.

pH is an important parameter that affects the photodegradation performance. To discuss its impact on the photodegradation reaction, we studied the photodegradation behavior of MB in phosphate buffer solutions with different pH values (Fig. S3†). The results showed that when the pH was between 5 and 8, as the pH increased, the photodegradation rate of methylene blue first increased and then decreased, reaching the maximum value when the pH was 7. This might be because under acidic conditions, the surface of BTO carried more positive charges, which generated electrostatic repulsion with the positively charged methylene blue molecules, resulting in a decrease in the adsorption ability and further leading to a reduction in the photocatalytic degradation efficiency. Under alkaline conditions, Ag nanoparticles might undergo chemical reactions with substances in the surrounding environment, thus undergoing surface passivation, which reduced the photodegradation rate of methylene blue. Therefore, better photodegradation performance was achieved when the pH was 7.

We also conducted a study on the photostability of methylene blue (MB), and the results are shown in the Fig. S4.† It can be seen from the figure that the absorbance of MB did not change after 1 hour of illumination, which indicates that under the experimental conditions, illumination hardly caused photodegradation of MB.

The variation in absorbance at 663 nm over time was used to investigate the reaction order of MB photodegradation. The absorbance-time scatter plot presented in Fig. 8C indicates that although absorbance and time are negatively correlated, which is nonlinear, indicating that the reaction is not a zero-order reaction and its rate is dependent on the MB concentration. A subsequent scatter plot of −ln(*C*/*C*_0_) *versus* time, as shown in Fig. 8D, reveals a clear linear correlation between these two parameters, confirming that the reaction follows a first-order kinetics. The reaction rate constants for MB photodegradation catalyzed by BTO and BTO/Ag were determined to be 0.0128 min^−1^ and 0.0381 min^−1^, respectively. Under the experimental conditions, the reaction rate using BTO/Ag as the photocatalyst was 2.98 times faster than that of BTO, demonstrating that the modification with Ag effectively enhanced the photocatalytic performance of BTO.

In order to reduce excessive dye-dependency on the results, we conducted a photodegradation experiment using methyl orange (Fig. S5†). According to the experimental results, as time went on, the absorbance of methyl orange gradually decreased, indicating that BTO/Ag also has a photocatalytic effect on methyl orange. Therefore, the photodegradation performance of BTO/Ag is not dependent on MB.

In order to explore the mechanism of the photodegradation reaction, we added *tert*-butanol, disodium ethylenediaminetetraacetate, and benzoquinone respectively in the photodegradation experiment of MB to study the main factors of photodegradation (Fig. S6†). Among them, *tert*-butanol is a scavenger for hydroxyl radicals (˙OH), disodium ethylenediaminetetraacetate is a scavenger for holes (h^+^), and benzoquinone is a scavenger for superoxide radicals (˙O_2_^−^). After reacting for 30 minutes, disodium ethylenediaminetetraacetate and benzoquinone had almost no effect on the absorbance of MB, while *tert*-butanol significantly increased the absorbance of MB. This result indicates that the presence of *tert*-butanol reduced the photodegradation rate of MB. Therefore, hydroxyl radicals are the main cause of MB degradation.

To prove the stability of the material, we carried out recycling experiments. The results showed that in five recycling experiments (Fig. S7†), the degradation rate of MB by BTO/Ag within 30 minutes was 32% × (1 ± 2.6%). This indicates that the material has good stability.

**Fig. 9:**
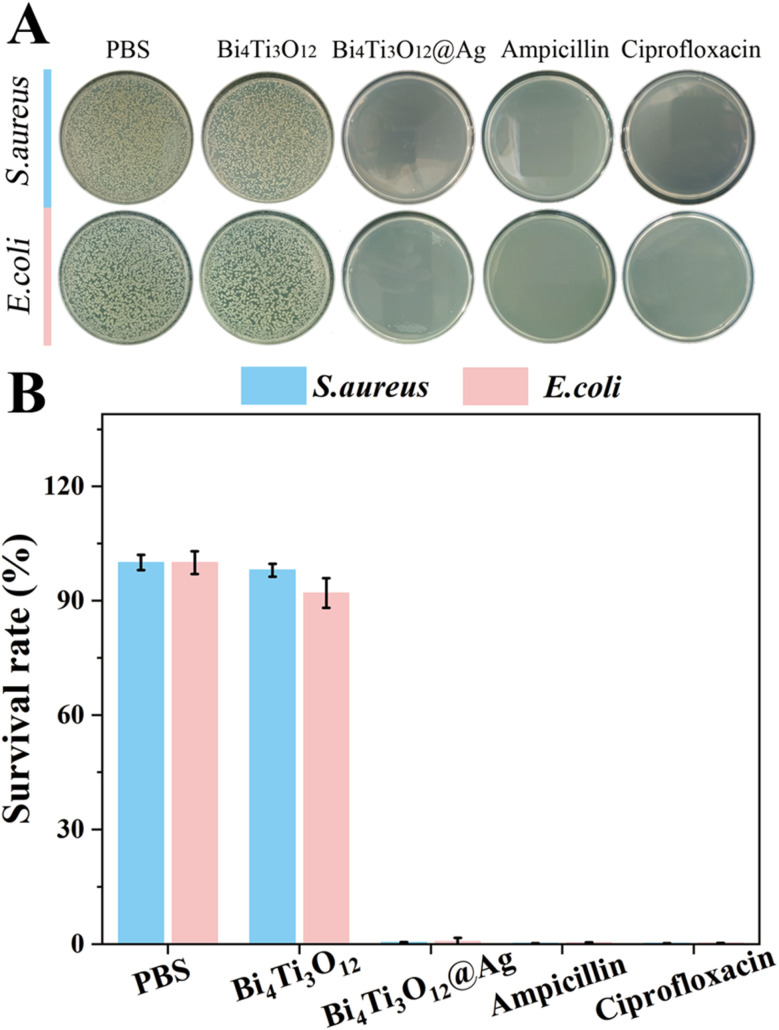
The (A) optical images and (B) statistical data of the antibacterial experimental results.

Considering that Ag is a well-known antibacterial material, this work also analyzed the antibacterial properties of BTO and BTO/Ag. *Escherichia coli* (*E. coli*) and *Staphylococcus aureus* (*S. aureus*), representing typical Gram-negative and Gram-positive bacteria, were selected for the experiments. Fig. 9A and B present the optical images and statistical data of the experimental results, respectively. It can be observed that BTO did not exhibit significant antibacterial effects against both bacterial strains. In contrast, BTO/Ag demonstrated remarkable antibacterial activity against both bacteria, with nearly zero bacterial survival rates in the experimental groups treated by BTO/Ag, which was the same as the experimental effect of ciprofloxacin at the same concentration. According to the above results, the performance of BTO/Ag is just average. Several factors contribute to this phenomenon: firstly, Ag possesses excellent antibacterial properties, which form the basis for the superior antibacterial performance of the as-prepared material. Secondly, BTO serves as an excellent carrier for Ag, enabling good dispersion of Ag without agglomeration during application, thereby further enhancing the antibacterial performance of BTO/Ag.

## 4 Conclusion

This study successfully synthesized BTO/Ag composites through a facile method by integrating BTO with Ag, and conducted a comprehensive analysis using various testing techniques. The SEM and TEM results revealed that BTO provided a suitable loading environment for Ag, enabling good monodispersity of Ag nanoparticles. The combined use of XPS, UV-Vis DRS, and EPR demonstrated that Ag optimized the electronic structure of BTO, resulting in strong UV light absorption capability, a narrower bandgap, and a higher oxygen vacancy concentration in BTO/Ag. EIS and M–S plot analyses indicated that Ag nanoparticles effectively enhanced the electron transfer capability and carrier density of BTO, resulting in substantial improvements in photocatalytic performance. In specific performance studies, ascorbic acid was used to evaluate photoelectrochemical sensing properties, MB (Methylene Blue) was examined for photodegradation performance, and *Escherichia coli* and *Staphylococcus aureus* were selected as representatives of Gram-negative and Gram-positive bacteria, respectively, for antibacterial performance research. The experimental results showed that BTO/Ag exhibited excellent properties in the fields of photoelectrochemical sensors, photodegradation, and antibacterial applications. In summary, BTO/Ag, as a multifunctional biomaterial, displayed outstanding performance and potential applications such as photoelectrochemical sensors, photodegradation, and antibacterial treatments.

## Data availability

The data supporting the findings of this study are available within the article.

## Conflicts of interest

There are no conflicts of interest to declare.

## Supplementary Material

RA-015-D4RA07385A-s001
