# Supplementary material for: Facile preparation of multifunctional biomaterials BTO/Ag and their applications in photoelectrochemical sensing, photodegradation and antibacterial activities
Source: RSC Adv. 2025 Jan 27;15(4):2657–67. doi: 10.1039/d4ra07385a (PMC11770675; doi:10.1039/d4ra07385a)
Supplement: RA-015-D4RA07385A-s001 [file RA-015-D4RA07385A-s001.pdf]

Supplementary Information (SI) for RSC Advances.
This journal is © The Royal Society of Chemistry 2025

Supporting Information.

Facile Preparation of Multifunctional Biomaterials BTO/Ag and Their Applications in Photoelectrochemical Sensing, Photodegradation and Antibacterial Activities

Zhang Kexi[a], Yan Bingdong[b], Chen Delun[a], Wang Xiaohong[a], Cao Yang[a,b], Zhang Xuewei[a,*], Hao Wanjun[a, *], Tu Jinchun[a,*]

[a] College of Material Science and Engineering, Key Laboratory of Advanced Materials of Tropical Island Resources, Ministry of Education, Hainan University, Haikou 570228, China

[b] Key Laboratory of Child Cognition and Behavior Development of Hainan Province, Qiongtai Normal University, Haikou, 571100, China

* Corresponding author

E-mail addresses:

zhangxuewei@hainanu.edu.cn (Zhang Xuewei);

hwj8899@hainanu.edu.cn (Hao Wanjun);

tujinchun@hainanu.edu.cn (Tu Jinchun).

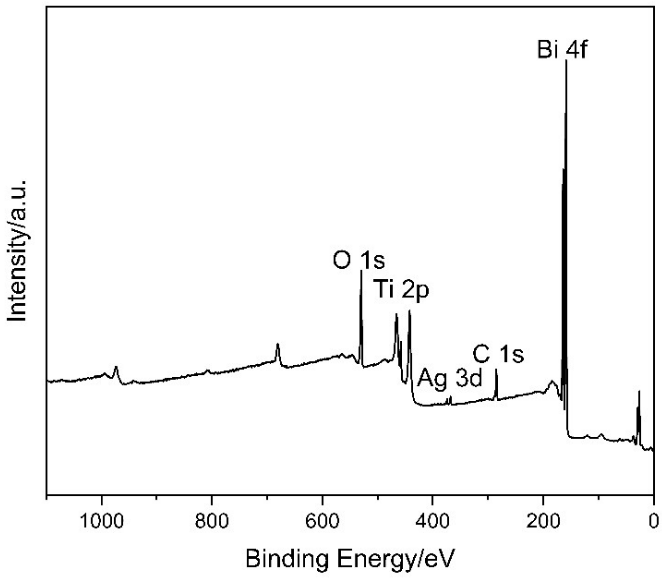

Figure S1 XPS survey spectra of BTO/Ag

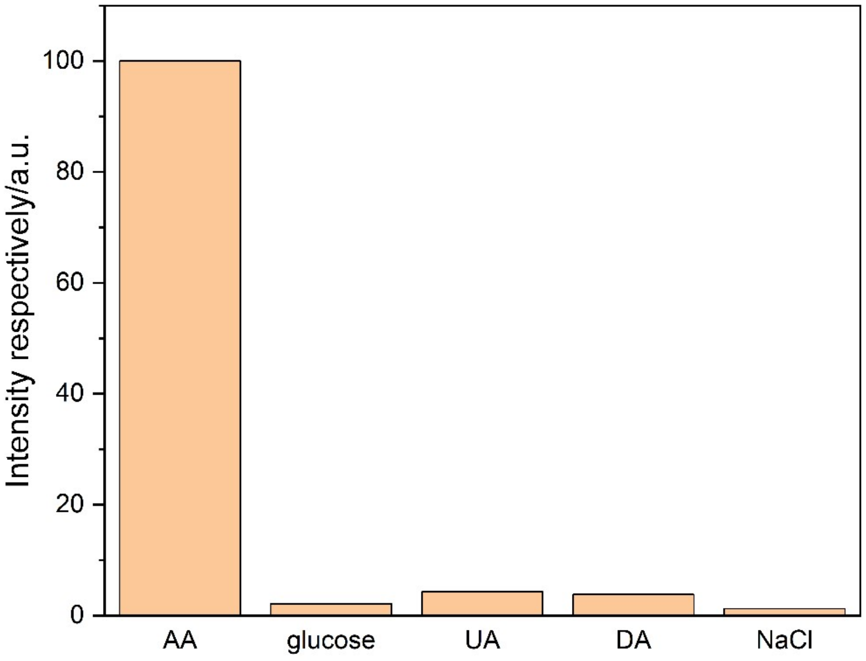

Figure S2 Results of the anti-interference experiment.

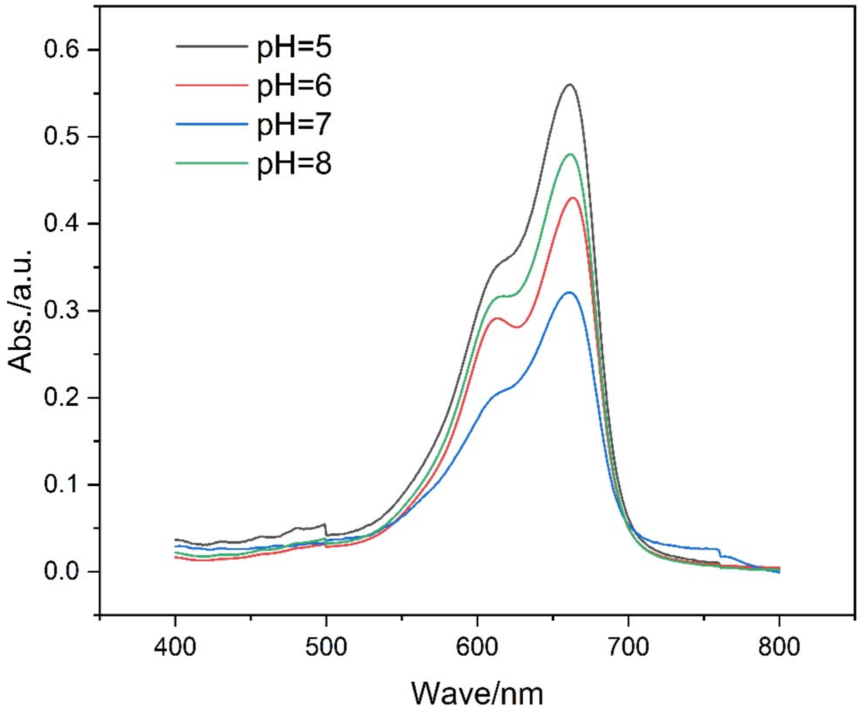

Figure S3 Photodegradation of MB under different pH values.

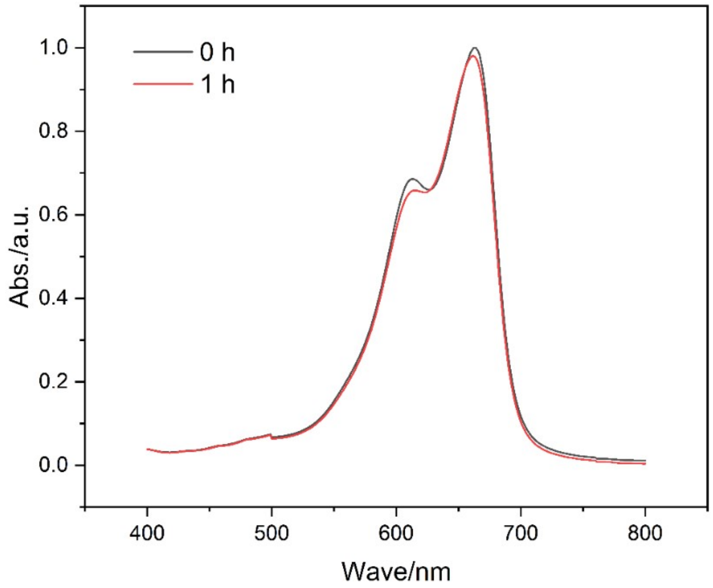

Figure S4 The impact of illumination on MB.

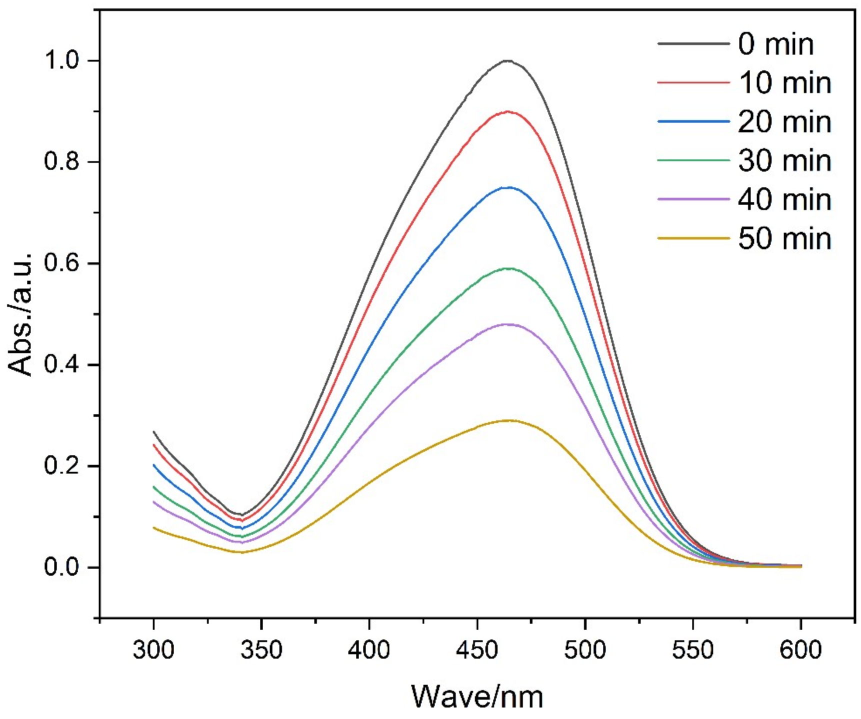

Figure S5 Photodegradation of methyl orange.

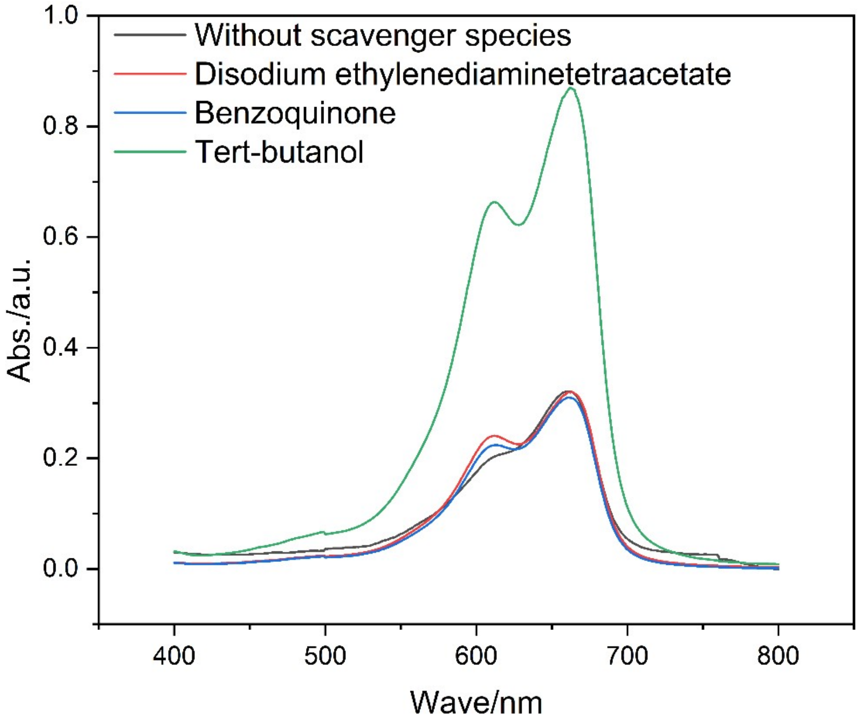

Figure S6 The effect of scavenger species on the photodegradation of MB.

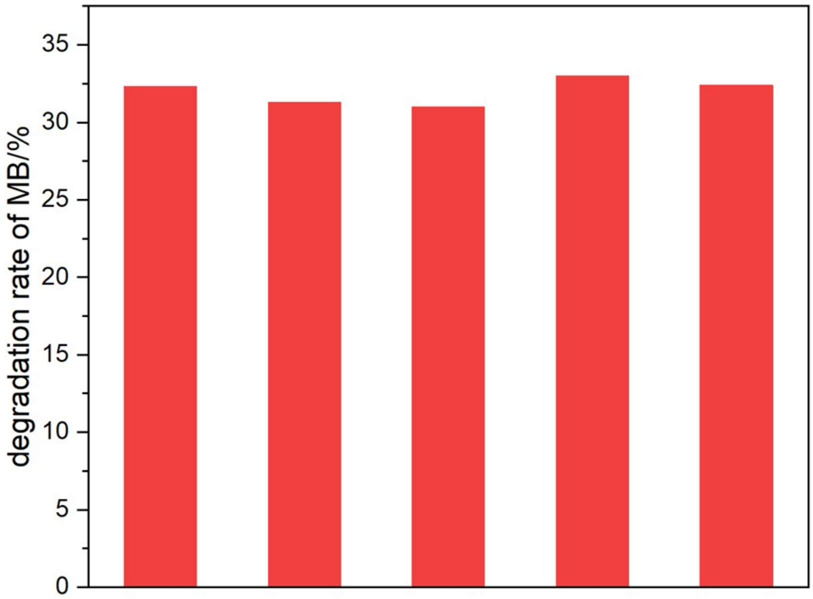

Figure S7 The results for individual recycling cycles toward the degradation rate of MB
